# Refractive Status at Birth: Its Relation to Newborn Physical Parameters at Birth and Gestational Age

**DOI:** 10.1371/journal.pone.0004469

**Published:** 2009-02-13

**Authors:** Raji Mathew Varghese, Vishnubhatla Sreenivas, Jacob Mammen Puliyel, Sara Varughese

**Affiliations:** 1 Department of Neonatology, St Stephens Hospital, Delhi, India; 2 Department of Biostatistics, All India Institute of Medical Sciences, New Delhi, India; 3 Christoffel-Blindenmission South Asia Regional Office, Delhi, India; University of Reading, United Kingdom

## Abstract

**Background:**

Refractive status at birth is related to gestational age. Preterm babies have myopia which decreases as gestational age increases and term babies are known to be hypermetropic. This study looked at the correlation of refractive status with birth weight in term and preterm babies, and with physical indicators of intra-uterine growth such as the head circumference and length of the baby at birth.

**Methods:**

All babies delivered at St. Stephens Hospital and admitted in the nursery were eligible for the study. Refraction was performed within the first week of life. 0.8% tropicamide with 0.5% phenylephrine was used to achieve cycloplegia and paralysis of accommodation. 599 newborn babies participated in the study. Data pertaining to the right eye is utilized for all the analyses except that for anisometropia where the two eyes were compared. Growth parameters were measured soon after birth. Simple linear regression analysis was performed to see the association of refractive status, (mean spherical equivalent (MSE), astigmatism and anisometropia) with each of the study variables, namely gestation, length, weight and head circumference. Subsequently, multiple linear regression was carried out to identify the independent predictors for each of the outcome parameters.

**Results:**

Simple linear regression showed a significant relation between all 4 study variables and refractive error but in multiple regression only gestational age and weight were related to refractive error. The partial correlation of weight with MSE adjusted for gestation was 0.28 and that of gestation with MSE adjusted for weight was 0.10. Birth weight had a higher correlation to MSE than gestational age.

**Conclusion:**

This is the first study to look at refractive error against all these growth parameters, in preterm and term babies at birth. It would appear from this study that birth weight rather than gestation should be used as criteria for screening for refractive error, especially in developing countries where the incidence of intrauterine malnutrition is higher.

## Introduction

Full term newborn babies are known to be on average hypermetropic at birth [1], [2], [3], [4]. Preterm babies tend to be myopic when examined at an age corresponding to term and later [5], [6], [7], [8]. A longitudinal study of 68 preterm babies reported that preterm babies were myopic to start with and became hypermetropic by 52 weeks [9]. We have previously reported refractive error at birth and its relation to gestational age [10]. This study had shown that preterm babies have myopia which decreases as gestational age increases.

In developing countries a large proportion of low birth weight babies (LBW: birth weight less than 2500 gms) may be small for gestational age (SGA). If refractive status is related primarily to gestational age, LBW babies could be expected to have a lower incidence of refractive error, as many of the LBW babies are not premature. In the present study, we have looked at the correlation between refractive error and birth weight, head circumference and length of the baby as well as the gestational age. We hypothesized that physical characteristics of the eye at birth, namely the size of the globe, the curvature of the cornea and lens characteristics, and therefore, the refractive error, may be correlated to physical characteristics like weight, length and head circumference more closely than with gestational age. To test this hypothesis we revisited the data on refractive error at birth [10].

## Materials and Methods

Of the 603 neonates in the original study [10], 44 babies could not be included because of the absence of data on one or more of the parameters being investigated in this study. In this analysis, data from 1118 eyes in 559 babies is analyzed. All babies delivered at St. Stephens Hospital and admitted in the nursery between June 2001 and September 2002, were eligible for the study. Informed consent was taken from the parents of subjects who were involved in the study. The study had the approval of the hospital research review board.

The weight of the newborn at birth was measured on an electronic weighing machine accurate up to 10 g. Gestational age was determined from the date of last menstrual period (LMP). If this was not known then gestational age determined by the first ultrasound was considered and if this too was unavailable then gestational age was determined using the New Ballard Score [11]. Babies born before 37 completed weeks of gestation were identified as preterms while those born after 37 completed weeks of gestation were taken as term babies. The length of the neonate was measured on an infantometer usually on the first day, or as soon as the condition of the baby was stable, in the first week of life. On the same day, head circumference was measured as the occipito-frontal circumference with non-elastic flexible tape (accurate to 0.1 cm) using the cross over technique. The instruments used, namely the electronic weighing machine, the infantometer and flexible measuring tape were not branded but generic instruments regularly used within the unit and tested for accuracy.

We studied the data from the right eye of each child for correlation of refractive status with gestation, length, head circumference and weight. The difference in MSE between the right and left eye was also studied to look for anisometropia at different gestational age, length, weight and head circumference. The method of testing refractive error has been previously reported in detail [10]. Briefly, refraction was performed within the first week of life by streak retinoscopy using a hand-held lens (without the use of a speculum). For cycloplegia and paralysis of accommodation, 0.8% tropicamide with 0.5% phenylephrine eye drops was used twice, one drop in each eye, at an interval of 15 minutes. Eyelids were separated manually without exerting pressure on the eye. Several readings were taken for each infant to look for variability of retinoscopy reflex due to residual accommodation. The figures were noted only after it was seen that there was no variation in this reading. The mean spherical equivalent (MSE) (spherical error plus half the astigmatic error) is commonly used to designate refractive error and this was studied against gestational age, birth weight, length and head circumference. Astigmatism was studied separately. We also looked at anisometropia (>1 dioptre difference of mean spherical equivalent between right and left eye).

Statistical analysis: The mean values, standard deviation, medians, range, and confidence intervals are reported. Simple linear regression analysis was performed to analyze the association of refractive error, mean spherical equivalent (MSE), astigmatism and anisometropia with each of the study variables, namely gestation, length, weight and head circumference. Multiple linear regression was carried out to identify the independent predictors for each of the outcome parameters. The relationship of the study variables (gestation, length, weight and head circumference) to the three categories of astigmatism (with the rule, against the rule and no astigmatism) were examined with one-way analysis of variance technique followed by Bonferroni adjustment for multiple comparisons. All statistical analyses were carried out using Stata 9.1 (Stata Corporation LP, 4905 Lakeway Drive, College Station, TX 77845, USA).

## Results

The various characteristics of the 559 newborns studied are shown in Table 1. It can be seen that there is a clear trend of increasing MSE and astigmatism (both mean and median) with an increase in each of the four study variables. For example, babies of 24–27 weeks gestation had a mean MSE of −2.79 dioptres (IQR −6 to +1.5), which gradually increased to 3.95 dioptres among babies with a gestation of ≥37 weeks (IQR +2.0 to +6.0). Similarly, median astigmatism increased from 0.0 dioptres in babies belonging to the lowest length group to 2.0 dioptres in babies with the highest length group. No such pattern could be seen with anisometropia.

**Table 1:** Refractive status (MSE, astigmatism and anisometropia) against growth parameters (weight, length and head circumference) and gestational age – Right Eye.

Characteristic	Number	MSE		Astigmatism		Anisometropia	
	(N = 559)	Mean (SD)	Median (IQR)	Mean (SD)	Median	Mean (SD)	Median
Gestation (weeks)							
24–27	7	−2.79 (3.92)	−5.5 (−6.0 +1.5)	1.00 (1.41)	0	1.14 (0.86)	1
28–30	42	+0.29 (3.55)	+0.5 (−2.25 +2.25)	1.24 (1.53)	0.5	1.01 (1.13)	1
31–33	98	+1.38 (3.36)	+1.5 (−0.5 +3.5)	1.50 (1.34)	1.2	1.14 (1.40)	0.5
34–36	156	+2.80 (3.37)	+3 (+1.0 +5.9)	1.54 (1.48)	2	1.26 (1.13)	1
37+	256	+3.95 (2.76)	+4 (+2.0 +6.0)	1.70 (1.59)	2	1.14 (1.16)	1
Weight (gms)							
<1000	18	−1.76 (4.23)	−3.5 (−5.1 +1.3)	1.19 (1.38)	0.5	1.1 (1.11)	1
1001–1500	81	+0.69 (3.52)	+0.5 (−2.0 +3.5)	1.03 (1.32)	1	1.14 (1.15)	1
1501–2000	158	+2.05 (3.18)	+2.0 (+0.5 +4.0)	1.65 (1.39)	2	1.21 (1.28)	1
2001–2700	140	+3.50 (2.77)	+3.5 (+2.0 +5.9)	1.76 (1.73)	2	1.20 (1.12)	1
2701+	162	+4.55 (2.60)	+5.0 (+3.0 +6.0)	1.65 (1.48)	2	1.10 (1.20)	1
Head circum. (cm)							
< = 25.0	16	−1.08 (4.47)	−2.5 (−4.9 +2.6)	0.91 (1.27)	0	1.05 (1.30)	1
25.1–28.0	59	+0.44 (3.61)	+0.5 (−2.0 +3.0)	1.30 (1.44)	1	1.31 (1.42)	1
28.1–30.0	93	+1.79 (3.20)	+2.0 (0.0 +4.0)	1.41 (1.72)	1	1.23 (1.19)	1
0.1–32.5	149	+2.48 (2.95)	+2.5 (+0.8 +4.5)	1.68 (1.55)	2	1.13 (1.17)	1
>32.5	242	+4.26 (2.85)	+4.2 (+2.5 +6.0)	1.68 (1.42)	2	1.13 (1.15)	1
Length (inches)							
< = 13.5	13	−2.06 (4.23)	−4.0 (−5.8 +2.1)	0.73 (1.05)	0	0.87 (1.14)	0.5
13.6–15.0	29	−0.34 (3.53)	−0.5 (−3.5 +2.2)	1.10 (1.42)	0	1.05 (0.97)	1
15.1–16.5	67	+1.06 (3.27)	+1.0 (−1.0 +3.5)	1.47 (1.47)	1	1.59 (1.33)	1
16.6–18.0	132	+2.48 (3.18)	+2.25 (+0.5 +5.0)	1.50 (1.41)	1	1.10 (1.13)	1
18.1–21.5	318	+3.82 (2.91)	+4.00 (+2.0 +6.0)	1.71 (1.57)	2	1.12 (1.19)	1

SD Standard deviation.

IQR Inter Quartile Range.

The pattern of astigmatism (with the rule, against the rule and no astigmatism) is observed to be similar (exact p = 0.26) in these two groups of term and preterm babies Table 2.

**Table 2:** Prevalence of astigmatism (1.00D or more) among term and preterm babies (Right Eye).

Type of astigmatism	Total	Preterm	Term
	(N = 559)	(N = 303)	(N = 256)
With the rule	309 (55.3%)	158 (52.1%)	151 (59.0%)
Against the rule	64 (11.4%)	38 (12.5%)	26 (10.2%)
No astigmatism	186 (33.3%)	107 (35.3%)	79 (30.9%)

Fisher's Exact p between Term and Preterm = 0.26.

**Figure 1:**
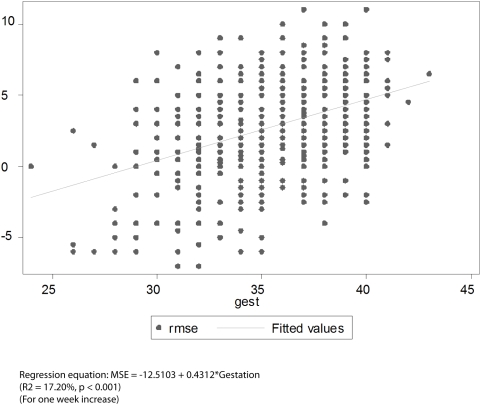
Association between MSE and Gestation (Right Eye).

Figures 1 to [Fig pone-0004469-g002] [Fig pone-0004469-g003] 4 depict the association of MSE to the various parameters: gestation, length, head circumference and weight.

**Figure 2 pone-0004469-g002:**
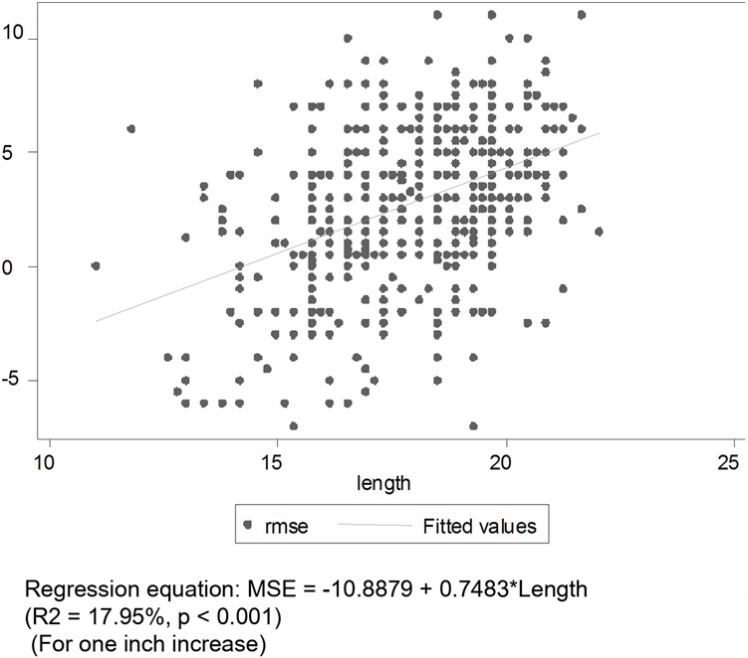
Association between MSE and Length (Right Eye).

**Figure 3 pone-0004469-g003:**
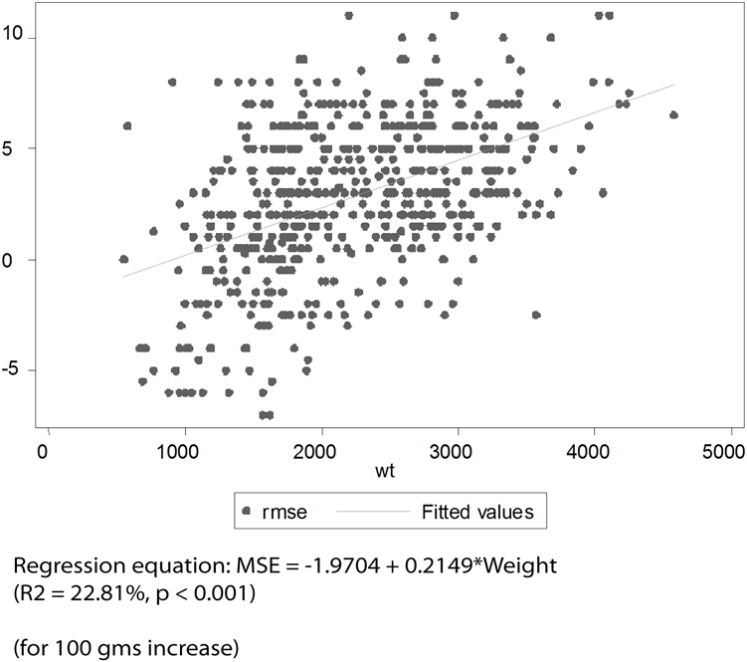
Association between MSE and Weight (Right Eye).

**Figure 4:**
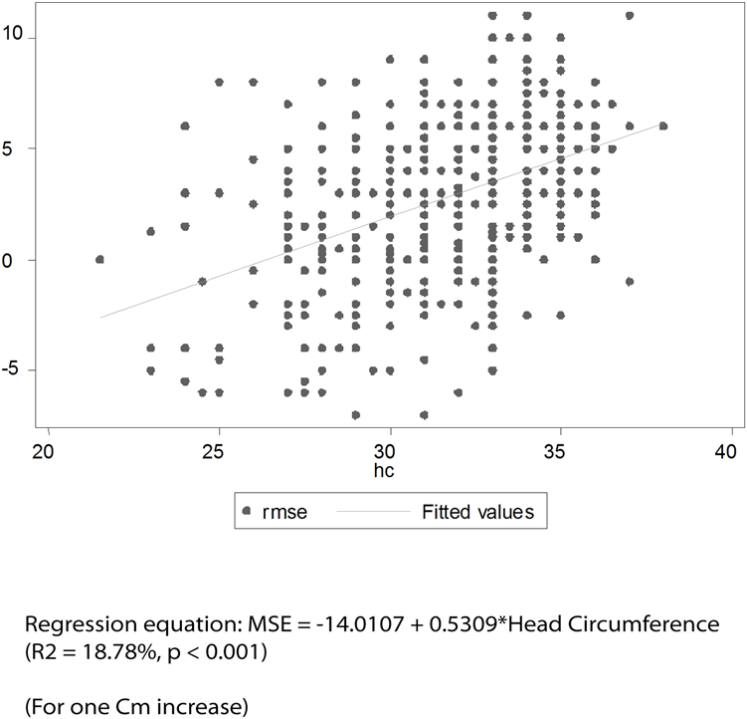
Association between MSE and Head Circumference (Right Eye).

Table 3 shows the correlation matrix of the 4 variables, gestation, weight, length and head circumference, to help understand the variable association of MSE to each of the parameters.

**Table 3:** Correlation matrix for the 4 variables gestation, weight, length and head circumference at birth (N = 559 babies).

Variable	Gestation	Weight	Length	Head Circumference
Gestation	1.00	0.75*	0.74*	0.74*
Weight		1.00	0.84*	0.84*
Length			1.00	0.80*

* signifies p<0.001.

Multiple linear regression analysis identified two independent predictors, namely birth weight and gestation, for MSE. Together they accounted for about a quarter of the variation in MSE. To understand further as to which of the two is more important, the mean MSE values were looked at separately for the various strata formed by gestation and weight (Table 4). It is clear that for any birth weight group, the MSE across the gestation groups are not very different as compared to an increasing trend of values across weight groups for any gestation group, suggesting that birth weight has a more important role than gestation. This is also confirmed by the partial correlations shown below Table 4. While the Pearson's correlations are similar, the partial correlation of weight with MSE, adjusted for gestation is more than the partial correlation of gestation with MSE, adjusted for weight (0.28 versus 0.10), though both the partial correlations are statistically significant.

**Table 4:** MSE (Right eye, N = 559) for various Gestation (in weeks) - Birth weight (g) groups with partial correlation and Pearson correlation of weight and gestation.

	< = 1500 g	1501–2000 g	2001–2700 g	2701+ g
24–27 Weeks	−2.79	—	—	—
28–30 Weeks	−0.24	1.35	—	—
31–33 Weeks	0.24	1.88	2.69	—
34–36 Weeks	1.37	2.15	3.70	4.62
37+ Weeks	2.88	2.41	3.40	4.56

Values are mean MSE in each combination group of gestation and birth weight.

Pearson Correlation of MSE with weight 0.48 (p<0.001) and with gestation 0.41 (p<0.001).

Partial Correlation of MSE with weight 0.28 (p<0.001) and with gestation 0.10 (p = 0.02).

None of the variables other than length was significantly associated with astigmatism in the multiple regression analysis.

## Discussion

This is the first study to look at refractive error against all these growth parameters, in preterm and term babies at birth. Our paper shows that the degree of hypermetropia decreases with increasing degree of prematurity with myopia noted in babies below 28 weeks of gestation. There was however only a small no. of preterms under 28 weeks of gestation in our study. To draw a more definite conclusion for this particular age group, a study looking at a much larger number is needed. In the present paper, we have looked at the correlation of refractive errors with birth weight, length and head circumference, in the first week of life among preterm and term newborn babies. In a developing country, there is both a higher incidence of preterm birth (due to poor antenatal care) and low birth weight (due to fetal under nutrition) [12]. We found that refractive error (MSE) correlates better with birth weight more than it did to gestational age. There have been few large studies looking at the refractive error in premature babies soon after birth. Most studies looking at refractive error in term and preterm babies have either involved a small number of subjects [8], or refraction was done at term or later [13]. A study on preterms from 2 weeks to 6 months of age from Israel reported no correlation of refractive error to gestational age or birth weight [14]. It is possible that emmetropization occurs and refraction studies done later, miss this initial refractive error. However, some authors suggest that emmetropization with age is not often complete and the initial refractive error during the critical phase of visual development may be one of the factors contributing to the high incidence of poor visual function found later in life in low birth weight children [8], [13], [14]. It has been suggested that the most important factor in the postnatal emmetropization of spherical equivalent refractive error is the modulation of axial growth in relation to the initial refractive error [15]. Refraction at a later age may underestimate the refractive error present at birth. Three studies from Israel have looked at refraction at birth against birth weight in preterm babies [16], [17], [18]. 54% of myopic preterms remained myopic when followed up to 7 years of age, though to a lesser degree [17]. The correlation between newborn length at birth and head circumference and refractive error at birth has not been examined previously. We found marked anisometropia, with over 30% babies having a difference of more than 1 diopter between the two eyes (This was seen across all gestations). No correlation of astigmatism with birth weight, length or head circumference was found in this study.

Cyclopentolate 0.5% is a better cycloplegic agent and has indeed been used in other studies. However, it is found to produce poor dilatation in pigmented irides and needs repeated instillation with the attended higher risk of gastric atony. It was for this reason, tropicamide and phenylephrine was used in our study. Residual accommodation, if any, with the agents we used would have resulted in a variability of the retinoscopy reflex but this was not found in a pilot study.

The findings in our study need to be corroborated by findings in other populations. The need for follow up of premature babies for refractive error is well established [19]. The study by Verma et al on 50 preterm infants showed none of the infants had normal vision at 6 months, and 16% had myopia while 20% had hypermetropia at 1 year. An inverse relationship was noted between gestational age and incidence of refractive error. Incidence of myopia was also shown to increase with decreasing weight. It would appear from our study that birth weight rather than gestational age should be used as a criteria for screening for refractive error.
